# Healthcare associated bloodstream infections – secular trends of 8 years hospital-wide surveillance in a tertiary care university hospital

**DOI:** 10.1186/1753-6561-5-S6-P238

**Published:** 2011-06-29

**Authors:** M-N Chraiti, W Zingg, V Sauvan, D Pittet

**Affiliations:** 1 Infection Control Programme, University of Geneva Hospitals, Geneva, Switzerland

## Introduction / objectives

Healthcare-associated bloodstream infection (BSI) is the 4^th^ major infection complication in medical care. BSI-surveillance is thought to be useful in monitoring trends of healthcare-associated infections (HAI) including outbreaks, emerging multiresistant pathogens, and effects of HAI intervention programmes.

## Methods

Introduced 1995 in acute care, prospective and systematic BSI surveillance at the University of Geneva hospitals was extended to a hospital-wide survey in 2003. Positive blood culture results identified by an electronic automated alert system were individually investigated and allocated to the following categories: contamination, secondary BSI (sBSI), primary BSI (pBSI) and catheter-related BSI (CRBSI).

## Results

A non-significant trend was detected for BSI to increase between 2003 (0.15 BSI/1000 patient-days) and 2009 (0.28/1000 patient-days) (IRR 1.09; 95%CI 0.73-1.62; p=0.67). This was predominantly due to rising rates of pBSI from 0.24/1000 patient-days in 2003 to 0.47/1000 patient-days in 2009 (IRR 1.05, 95%CI 0.62-1.77; p=0.85). No change was detected for sBSI and contaminations while CRBSI episodes increased between 2006 (0.17/1000 patient-days) and 2007 (0.27/1000 patient-days). All BSI-outcomes decreased in 2010 following a hospital-wide training in catheter care. No major outbreak was detected.

## Conclusion

Eight years BSI-surveillance did not show significant variation making this mode of surveillance less likely to be affected by confounding factors. Whether improved catheter care contributed to the reduced BSI-rates in 2010 needs to be confirmed.

## Disclosure of interest

None declared.

